# ST waveform analysis for monitoring hypoxic distress in fetal sheep after prolonged umbilical cord occlusion

**DOI:** 10.1371/journal.pone.0195978

**Published:** 2018-04-16

**Authors:** Peter Andriessen, Alex Zwanenburg, Judith O. E. H. van Laar, Rik Vullings, Ben J. M. Hermans, Hendrik J. Niemarkt, Reint K. Jellema, Daan R. M. G. Ophelders, Tim G. A. M. Wolfs, Boris W. Kramer, Tammo Delhaas

**Affiliations:** 1 Department of Pediatrics, Máxima Medical Centre, Veldhoven, the Netherlands; 2 Department of Pediatrics, Maastricht University Medical Centre, Maastricht, the Netherlands; 3 Department of Biomedical Engineering, Maastricht University, Maastricht, the Netherlands; 4 CARIM School for Cardiovascular Diseases, Maastricht University, Maastricht, the Netherlands; 5 School for Mental Health and Neuroscience, Maastricht University, Maastricht, the Netherlands; 6 Department of Obstetrics and Gynecology, Máxima Medical Centre, Veldhoven, the Netherlands; 7 Signal Processing Systems group, Department of Electrical Engineering, Eindhoven University of Technology, Eindhoven, the Netherlands; University of Washington, UNITED STATES

## Abstract

**Introduction:**

The inconclusive clinical results for ST-waveform analysis (STAN) in detecting fetal hypoxemia may be caused by the signal processing of the STAN-device itself. We assessed the performance of a clinical STAN device in signal processing and in detecting hypoxemia in a fetal sheep model exposed to prolonged umbilical cord occlusion (UCO).

**Methods:**

Eight fetal lambs were exposed to 25 minutes of UCO. ECG recordings were analyzed during a baseline period and during UCO. STAN-event rates and timing of episodic T/QRS rise, baseline T/QRS rise and the occurrence of biphasic ST-waveforms, as well as signal loss, were assessed.

**Results:**

During baseline conditions of normoxemia, a median of 40 (IQR, 25–70) STAN-events per minute were detected, compared to 10 (IQR, 2–22) during UCO. During UCO STAN-events were detected in five subjects within 10 minutes and in six subjects after 18 minutes, respectively. Two subjects did not generate any STAN-event during UCO. Biphasic ST event rate was reduced during UCO (median 0, IQR 0–5), compared to baseline (median 32, IQR, 6–55). ST-waveforms could not be assessed in 62% of the recording time during UCO, despite a good quality of the ECG signal.

**Conclusions:**

The STAN device showed limitations in detecting hypoxemia in fetal sheep after prolonged UCO. The STAN device produced high false positive event rates during baseline and did not detect T/QRS changes adequately after prolonged fetal hypoxemia. During 14% of baseline and 62% of the UCO period, the STAN-device could not process the ECG signal, despite its good quality. Resolving these issues may improve the clinical performance of the STAN device.

## Introduction

During labor and delivery, umbilical cord blood flow may be disturbed by uterine contractions. The temporary reduction in blood flow and oxygen delivery induces changes in fetal blood pressure and oxygen pressure [1]. Cardiotocography (CTG) is used to identify fetuses in hypoxic distress. The introduction of continuous CTG monitoring during labor led to an increase in operative deliveries [2]. The use of CTG reduced the risk for developing seizures, but had no effects on perinatal mortality or the incidence of cerebral palsy [2]. Therefore, additional markers for monitoring fetal distress have been developed. The ST interval of the fetal electrocardiogram (ECG) represents the energy-consuming process of ventricular repolarization. The interval between the ends of the QRS-complex and the T-wave represents the process of ventricular repolarization [3–5]. Because changes in the T wave amplitude of the fetal ECG were found to be associated with hypoxic distress [6–8], an ST waveform analysis (STAN) algorithm and device was developed, and introduced in many clinics in addition to CTG registration. However, recent clinical trials and meta-analyses, which evaluated the effect of STAN in addition to CTG, were unable to show conclusive benefits of STAN on perinatal outcomes or caesarean section rates [9–12]. Because ST events occur in a similar percentage in normal, intermediary and abnormal CTG recordings, STAN remains dependent on CTG analysis [12]. However, the large intra- and inter-variability of the CTG interpretation hampers good clinical judgment for intervention [13].

In addition, the STAN monitor may not be sufficiently sensitive to detect hypoxic distress at an early stage. If a STAN-event is detected too late, even with rapid intervention, hypoxic distress may have already resulted in irreversible brain injury. Early warning is thus essential to improve perinatal outcomes. Therefore, both false positive and false negative STAN-events may limit the clinical performance of the STAN device.

Fundamental experimental evaluation of the STAN monitor performance has not been conducted and may identify points for improvement of the technique. Measures of tissue perfusion (e.g. blood pressure, blood gas measurements) and continuous ECG recordings are required to establish fetal hypoxic distress against which the STAN performance can be compared. Therefore, the aim of the study was to assess STAN-events with respect to rates and timing using a standardized preterm ovine model for global hypoxia-ischemia. Although the preterm ovine fetus is more resistant to hypoxic distress than the term ovine fetus, the ST waveform response to hypoxic distress is comparable [14, 15]. After a normoxic period of four hours, severe hypoxic distress was induced in the model by a transient umbilical cord occlusion (UCO) of 25 minutes. To enable cross-referencing output of the STAN device with the original fetal ECG, an external measurement device recorded the fetal ECG during both normoxemia and periods of hypoxemia. Subsequently, the recorded ECG was imported to a STAN device to assess event rate and timing of STAN-events.

## Materials and methods

### Animal experiment

The study group consisted of eight singleton fetuses of both sexes from time-mated Texel ewes, randomly drawn from a larger cohort of fetal sheep, which was measured over the period February 2010 to November 2013 [16–18]. The original cohort studies—set up to investigate treatment of preterm hypoxic-ischemic brain injury—were conducted at the Maastricht University Medical Centre, Maastricht, the Netherlands. The experimental protocol and design of the studies were in line with the institutional guidelines for animal experiments and were approved by the institutional Animal Ethics Research committee of Maastricht University, The Netherlands. Use of this data for the current study neither affected measurement protocol nor study setup of the original studies since the animal operators were blinded.

Fetuses were instrumented at 101.7 ± 1.0 (mean ± SD) days gestational age (full gestation equals 146 days). Anesthesia was performed as previously described [16]. In short, anesthesia was induced by thiopental (15 mg/kg) intravenously. After intubation, general anesthesia was maintained with 1 to 2% isoflurane guided by depth of sedation and supplemented by remifentanil (0.75 μg/kg/minute) intravenously for analgesia. Vital parameters and depth of sedation were continuously monitored by certified personnel. Instrumentation was performed according to the procedure described previously by Jellema et al [16]. During instrumentation three ECG electrodes (5 mm; Cooner Wire Co., Chatsworth, CA) were fixed subcutaneously with sutures to form the Einthoven ECG leads I, II and III. An inflatable occluder (OC16HD, In Vivo Metric, Healdsburg, CA) was placed around the umbilical cord. A 3.5 French polyurethane umbilical vessel catheter (Tyco Healthcare Group, Mansfield, MA) was inserted into the femoral artery of the fetus to allow withdrawal of blood samples for blood gas analysis and for monitoring arterial blood pressure. Ewes had ad libitum access to water and food. The welfare of the animals was monitored daily by certified personnel. Ewe and fetus were allowed to recover for 3 to 4 days. Subsequently, the umbilical occluder was inflated for 25 minutes to induce transient severe global hypoxia-ischemia. A baseline period of four hours prior to UCO was chosen for the ECG analysis, to allow the STAN device to determine baseline settings.

### Data acquisition

Fetal arterial blood was sampled to measure arterial blood acidity level (pH), base excess, arterial partial oxygen pressure (PaO_2_) and arterial partial carbon dioxide pressure (PaCO_2_) on a i-STAT 1 Blood Gas Analyzer (Abbott Laboratories, Illinois, USA). Baseline samples were drawn 1 hour before UCO. During UCO, samples were drawn at 5, 10 and 20 minutes after start of the UCO. In the reperfusion period, samples were drawn at 5, 10, 20, and 30 minutes after occlusion end. After a reperfusion period of 7 days (according to the original cohort study protocol), animals were sacrificed with intravenous pentobarbitone (200mg/kg).

ECG data were acquired and digitized by an MPAQ unit (Maastricht-Programmable AcQuisition system, Maastricht Instruments BV, Maastricht, The Netherlands) at a sample frequency of 1000 Hz. Matlab 2013a (The MathWorks, Natick, MA, USA) was used for further processing and analysis of ECG recordings from leads I and II during the period from 4 hours before UCO and the subsequent 25 minutes of occlusion. Leads I and II were digitally projected to a negative aVF (-aVF) lead to reproduce the signal that would be measured using a unipolar scalp electrode. Subsequently, recordings were converted to an analog signal using the digital-analog converter on a Realtek ALC892 chipset (Realtek Semiconductor Corp., Taiwan). An analog oscilloscope was used to check whether the analog output signal matched that of the input ECG. The analog signal was offered real time to a Goldtrace fetal scalp electrode (FSE-8000G, Neoventa Medical AB, Mölndal, Sweden) connected to a STAN S31 fetal heart monitor (Neoventa Medical AB). The heart rate monitor provided real time display of heart rate and ST waveform analysis. ECG displayed by the heart rate monitor resembled clinical fetal ECG in shape and amplitude. Screen output was simultaneously printed to paper by STAN P11 printer (Neoventa Medical AB). Events and other log entries were printed after the end of the recording. Logs were entered manually into Microsoft Excel version 14.0.7151.5001 (Microsoft Corporation, Redmond, WA) for further analysis using Matlab.

### Data analysis

We refer to previous work of detecting the characteristic points, segments, and intervals of the ECG [19]. HR was detected from RR intervals and calculated as a mean HR (beats per minute, bpm). HR variability was calculated by determining the SD of RR intervals (SDNN, ms) of the previous minute. According to the STAN guidelines, the following ST waveform-based events were considered significant during the UCO (with associated changes in fetal HR) [4]. First, an episodic rise implicates that the T/QRS ratio rises and returns within 10 minutes. As the degree of change in T/QRS reflects fetal stress, an episodic T/QRS rise over 0.10 was considered significant. Second, a baseline rise means that the change in T/QRS ratio lasts for more than 10 minutes. A baseline T/QRS rise of more than 0.05 is regarded as significant and is indicated as an ST event. Third, biphasic ST waveforms that cut across baseline or are entirely below baseline are considered significant when they have lasted for more than two minutes or occur repeatedly. The event log of the STAN device provides information about the change and time of occurrence of the previously described events.

We used two approaches to analyze the performance of the STAN device. First, to determine the time until the first STAN-event during the hypoxic period, we constructed a detection curve for the events recorded during this period. Second, during the normoxic baseline and hypoxic periods the rate at which STAN-events were detected was determined.

Furthermore signal loss as reported through the log entries was analyzed. The STAN device distinguishes two types of signal loss, namely loss of ST waveforms and loss of fetal HR. Loss of ST waveforms signifies intervals where the STAN device could not assess the ST waveform but still could assess fetal HR. Signal loss due to loss of fetal HR indicates intervals where HR could not be determined by the STAN device. We cross-referenced HR signal loss events with the input ECG to specify causes of HR loss in the device. Four conditions were evaluated: disconnection of input ECG signal, HR above 230 bpm or below 50 bpm because at these heart rates the STAN device cannot determine HR, and unidentified loss of the HR signal.

Statistical analysis: Data with normal distribution were expressed as mean ± standard deviation (SD), otherwise as median and inter quartile range [IQR]. STAN-event rates were compared between normoxic and hypoxic periods using a Wilcoxon signed rank, and *p*<0.05 was considered significant.

## Results

**Fig 1:**
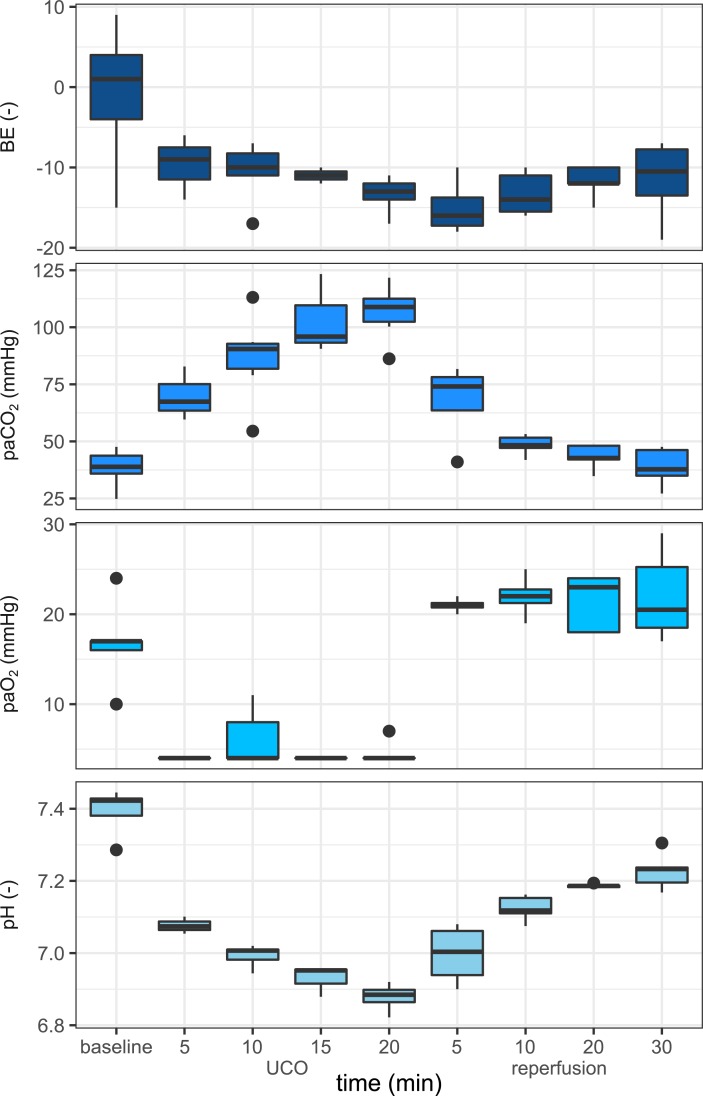
Blood gas analysis during normoxemia and hypoxemia. Whisker plots for arterial blood pH (panel A), base excess (panel B), partial arterial oxygen pressure (panel C) and partial arterial carbon dioxide pressure (panel D), with whiskers extending up to 1.5 times IQR. Blood gasses were assessed during baseline, during (5-10-15-20 minute) umbilical cord occlusion and during reperfusion. Outliers are displayed as circles.

**Fig 2:**
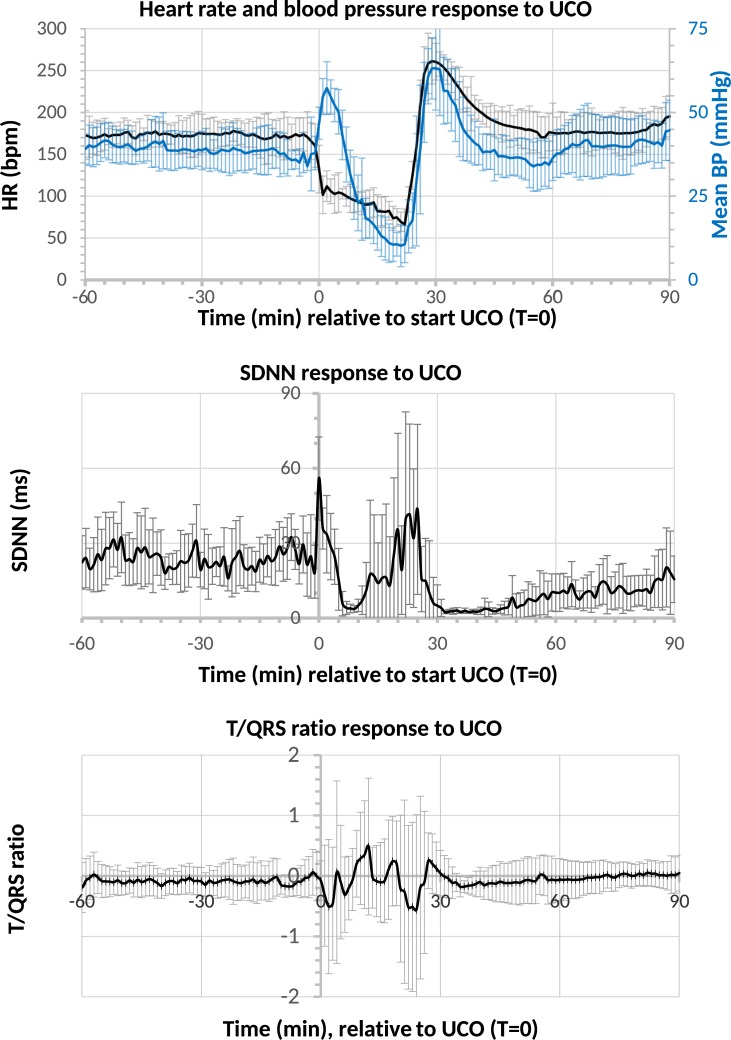
Blood pressure and heart rate response during normoxemia and hypoxemia. Mean arterial blood pressure (BP), heart rate (HR, beats per minute, bpm), heart rate variability (expressed as SDNN, ms) and T/QRS ratio during baseline, UCO (T = 0–25 minute) and reperfusion of all subjects. The mean values are shown with 95% confidence intervals. Note the marked bradycardia and initial hypertension directly after cord occlusion; during occlusion a sustained bradycardia and progressive hypotension was observed. After umbilical cord occlusion, the reperfusion period showed a marked decrease in SDNN compared to the baseline period.

Blood gas measurements confirmed the severe hypoxemia after UCO. PaO_2_ decreased from 17 ± 4 to 5 ± 1 mmHg, PaCO_2_ increased from 38 ± 8 to 107 ± 12 mmHg and arterial pH decreased from 7.40 ± 0.05 to 6.88 ± 0.03 at the end of UCO (**Fig 1**). Acidosis became more severe as UCO was prolonged, with a concomitant rise in PaCO_2_. Release of UCO was followed by an immediate increase in PaO_2_, a gradual increase in arterial pH and a gradual decrease in PaCO_2_. **Fig 2** shows grouped data of HR, SDNN and T/QRS ratio as calculated from the input ECG during the baseline, UCO and reperfusion phase. When comparing the baseline period (60 min before the umbilical cord occlusion) with the reperfusion period (30–90 minutes after start of the occlusion) a marked decrease in SDNN was observed. The baseline SDNN was 23.9 ± 3.7 ms whereas reperfusion SDNN was 6.1 ± 4.5 ms (Wilcoxon signed rank, p<0.001)”.

**Fig 3:**
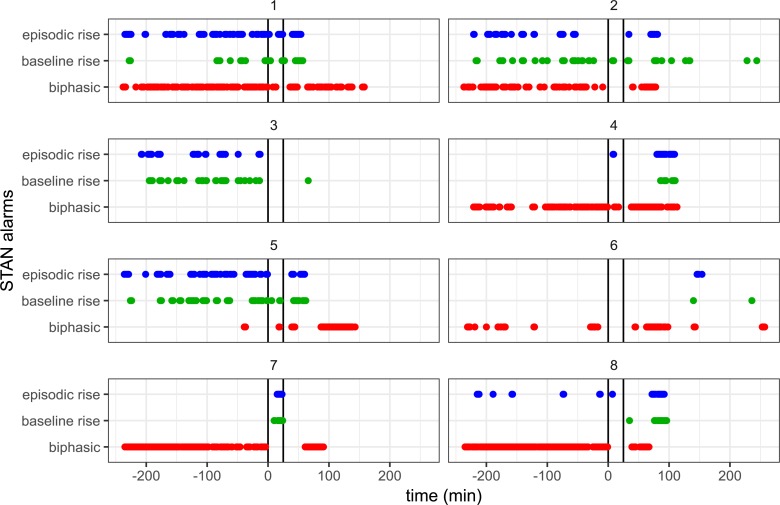
STAN-events during normoxemia and hypoxemia. Subjects with STAN-events in 1-minute windows during the 4 h baseline period and during the 25 minute transient umbilical cord occlusion (indicated by vertical black lines). The events assessed are episodic T/QRS rise (blue), baseline T/QRS rise (green) and biphasic ST events (red). The numbers refer to individual subject numbers.

STAN-events and corresponding event rates are shown in **Fig 3** and **Table 1**, respectively. Note that all subjects showed STAN-events during the baseline period. In general, event rates were high during the baseline period. The event rates during baseline and UCO were not significantly different for baseline and episodic T/QRS events, while biphasic ST event rate was significantly reduced (*p<*0.05) during UCO. During the hypoxic period, episodic T/QRS and baseline T/QRS events were detected in four subjects, while three subjects showed biphasic ST events. No STAN-events for hypoxic distress were detected in two subjects despite UCO.

**Table 1:** Event rates during baseline and fetal hypoxemia.

subjects	episodic T/QRS event		baseline T/QRS event		biphasic ST event	
	baseline	UCO	baseline	UCO	baseline	UCO
**1**	80	10	15	3	200	3
**2**	34	0	24	3	102	0
**3**	34	0	29	0	0	0
**4**	0	2	0	0	156	5
**5**	72	0	36	3	5	2
**6**	0	0	0	0	32	0
**7**	0	9	0	9	273	0
**8**	10	1	0	0	332	0
**events/h**	5.5 [0.0–10.9]	1.2 [0.0–9.0]	1.9 [0.0–6.3]	3.6 [0.0–7.2]	32.3 [6.3–54.6]*	0.0 [0.0–5.4]

Legend: Event rates during normoxemia (baseline) and hypoxemia (umbilical cord occlusion, UCO). Events are shown as absolute numbers for the baseline period (4 h) and UCO period (25 minute), respectively. The median [IQR] event rate/h is calculated from the averaged events/h over all subjects. Differences between baseline and UCO were assessed using the Wilcoxon signed rank test.

* p<0.05.

**Fig 4:**
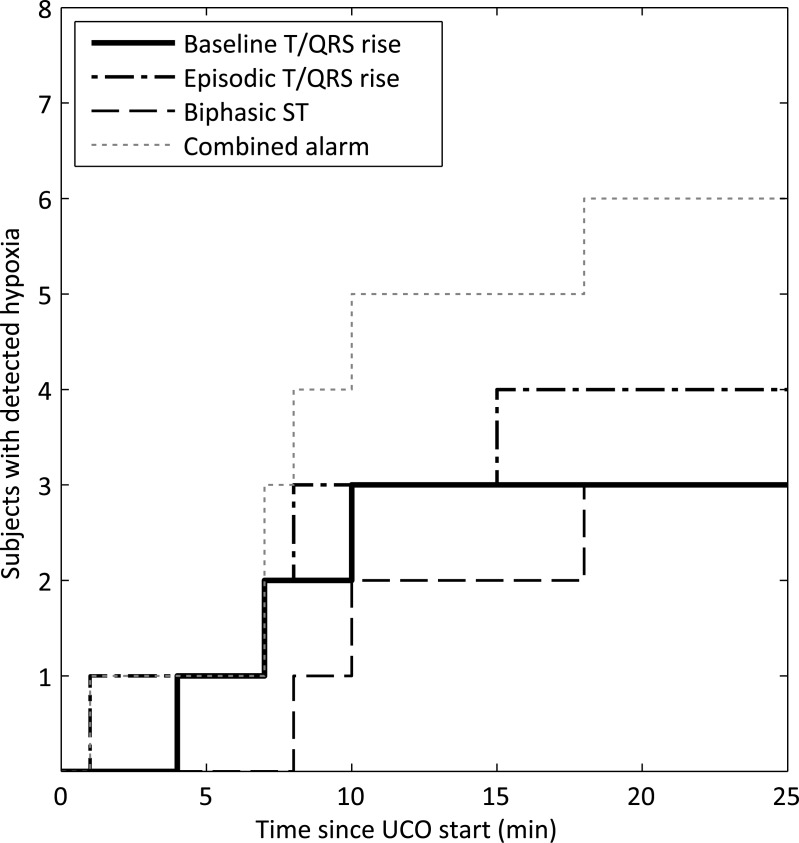
STAN-event warning during fetal hypoxia. First warning for hypoxic distress by the different STAN device events during the 25 minute umbilical cord occlusion. Time t = 0 refers to the start of the umbilical cord occlusion. In two subjects not any STAN-event was detected.

Event timing during UCO is shown in **Fig 4**. During fetal hypoxemia, events were detected in five subjects within 10 minutes and in six subjects within 18 minutes. No STAN-events were detected for the two remaining subjects.

**Fig 5:**
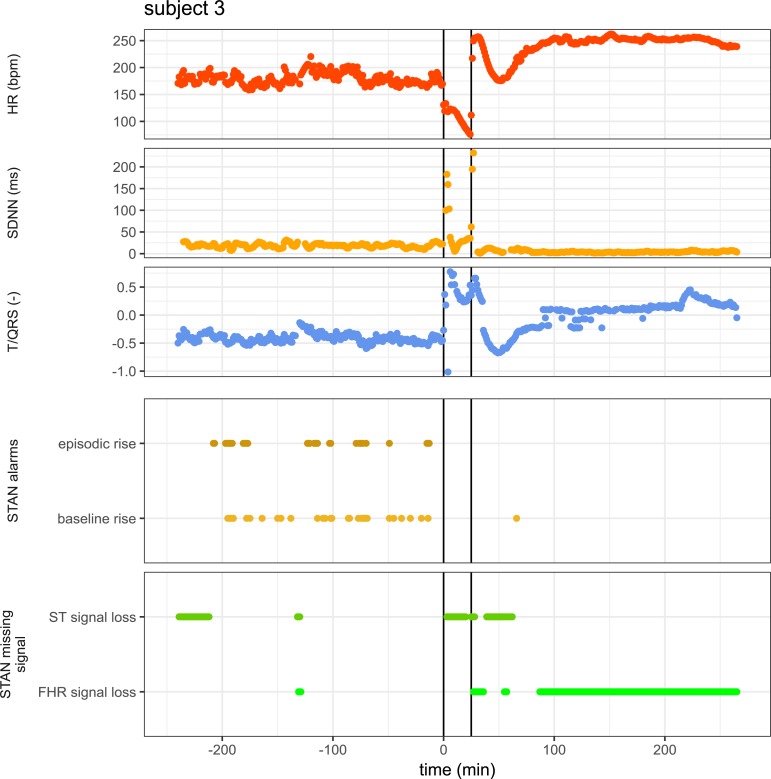
Example of failure of the STAN monitor to detect any ST-event during umbilical cord occlusion in subject 3. The top three panels show the calculated heart rate (HR), SDNN (estimate of HR variability) and T/QRS ratio from the fetal ECG as determined using our analysis technique as described [19]. The bottom two panels show STAN events and signal loss. The vertical lines indicate start and end of the umbilical cord occlusion. Note that during cord occlusion the STAN monitor indicated ST signal loss and no events were detected, despite calculated HR, SDNN and T/QRS ratio from the input ECG.

We analyzed why input ECG could not be assessed by the STAN device. In **Table 2** we present the results on the comparison between input ECG on the one hand, and ST and HR signal loss as indicated by the STAN device on the other hand. During the baseline period, i.e. the four hours prior to UCO, the STAN device could not assess the ST waveforms in 14% of the signal. During the 25 minutes of UCO, the STAN device could not assess ST waveforms in 62% of the time, which is significantly more than during baseline (*p<*0.05). In **Table 3** the data of missing (input) ECG signal is presented during baseline (median 1.7%, IQR 1.7–2.2) and UCO (median 0%, IQR 0–0). Of the two subjects (number 3 and 6) without any STAN-events during cord occlusion, only 2.5 and 1.7% during baseline and 0% and 8% of the input ECG during UCO was missing, respectively. **Fig 5** illustrates the failure of the STAN device to detect ST-events, despite ECG being present and adequate quality of the input ECG to calculate HR, HR variability and T/QRS.

**Table 2:** Signal loss in STAN recordings in all subjects.

	baseline	UCO
**STAN no ST waveform detection**	14% [5–20]*	62% [29–76]
**STAN no fHR detection**	1 [1–3]%	0 [0–7]%
• No ECG recording	1 [1–2]%	0 [0–0]%
• HR < 50 bpm	0 [0–0]%	0 [0–0]%
• HR > 230 bpm	0 [0–0]%	0 [0–0]%
• Undefined loss	0 [0–0]%	0 [0–6]%

Legend: STAN logs indicate segments where either ST waveforms cannot be detected or fetal heart rate (fHR) is lost. Cross-reference of the fHR with input ECG signal specifies four causes of fHR loss: absent recording (no ECG recording), severe bradycardia (HR < 50 bpm), severe tachycardia (HR > 230) and fHR loss that could not be attributed to the previous causes (undefined HR loss). Note that undefined fHR loss occurred despite the presence of ECG input signal. Values are percentage of loss relative to recording time, expressed as median [IQR] of eight subjects. Difference between baseline and UCO were assessed using the Wilcoxon signed rank test.

* indicate p<0.05.

**Table 3:** Missing input ECG signal in all subjects.

	Missing ECG signal (% of period)	
Subject number	Baseline (240 minutes)	UCO (25 minutes)
1	1,7%	0,0%
2	0.8%	0,0%
3	2,5%	0,0%
4	0,0%	0,0%
5	2,5%	0,0%
6	1,7%	8,0%
7	0,0%	0,0%
8	2,1%	0,0%

Legend: Missing (input) ECG signal, values are percentage of missing ECG signal to recording time.

## Discussion

Waveform changes in the ST segment of the ovine fetal electrocardiogram are considered to be a sensitive parameter of hypoxic stress, where the dominant response is a progressive increase in the T wave amplitude, and where the changes can be quantified as the T/QRS ratio [7, 20]. Although clinical guidelines for STAN were defined for term infants, preterm and term ovine fetuses showed hypoxia-induced ST waveform changes which were comparable to that of the human term infant [14, 15]. Also the fetal sheep model is regarded as a suitable model for studying fetal cardiovascular [21] and cerebral [22, 23] responses to hypoxia-ischemia. Therefore, we hypothesized that in a preterm fetal sheep model—exposed to prolonged UCO—a clear distinction between conditions of normoxemia and hypoxemia can be made, that on its turn allows the assessment of false and valid STAN-events. The main finding of the study is that the STAN device showed severe limitations in detecting hypoxemia in fetal sheep during prolonged UCO despite appropriate signal input. The STAN device produced high false positive event rates during baseline and did not detect T/QRS changes adequately after prolonged fetal hypoxemia.

The identified problems were paradoxically high event rates of episodic T/QRS, baseline T/QRS and biphasic ST during normoxemia, whereas in the 25 minutes following complete UCO the event rate of T/QRS changes were not different from baseline or even decreased, when compared to baseline. Moreover, despite UCO-induced hypoxemia, STAN-events were completely absent in two out of eight subjects. The absence of STAN-events during UCO could not be explained by the lack of signal quality during UCO as evidenced by the fact that despite the presence of a fetal ECG signal, the STAN device could not perform ST waveform analysis during 62% of the recorded time during hypoxemia. These findings indicate that high false event rates and loss of monitoring capacity during fetal hypoxic distress, potentially limits the clinical usefulness of STAN.

Surprisingly, ST events occurred during the baseline period more frequently than during hypoxemia. Similar results have been reported in human fetuses during labor [12]. In human fetuses, this may be explained by temporary hypoxemia due to uterine contractions during labor. However, our model shows that also in a baseline period (without labor and without short periods of hypoxemia) STAN-events occur very frequently. In other words, STAN-events may occur irrespective of fetal hypoxemia and associated ischemia. Therefore, one could question the value of episodic T/QRS rise, baseline T/QRS rise and biphasic ST waveforms as indicators of fetal hypoxemia and associated ischemia. In human fetuses, it has been shown that the presence of significant biphasic events did not discriminate in the prediction of fetal distress or adverse outcome [24]. In the current study none of the STAN-events was able to discriminate for fetal condition either.

STAN-events were detected in five subjects after approximately 10 minutes of fetal hypoxia. Remarkably, even after 25 minutes of severe hypoxemia no STAN-events were detected in two of eight subjects. The ‘late’ response and more so the ‘no event’ response are at odds with rapid intervention that is necessary to prevent major brain injury.

The current study suggests that the absence of STAN-events is not due to missing ECG input (Table 3) since the animals used in this study were also analyzed according to the approach described in a previous paper by which we calculated HR, SDNN and T/QRS values (Fig 2) [19]. Analogue to STAN device, episodic rise in T/QRS value was defined as a positive change between the actual T/QRS value and the median T/QRS value of the previous 10 minutes. Also analogue to STAN device alarms, baseline rise in T/QRS value was defined as a positive change from baseline by comparison of the median T/QRS value of the last 10 minutes with the baseline T/QRS value. With this approach we could calculate T/QRS values throughout each experiment and could detect episodic and baseline rise T/QRS changes from the fetal ECG during normoxemia and the period of UCO, even if the STAN-device was unable to do so (Figs 3 and 5). In summary, this indicates that the ECG waveform, though adequate for HR analysis, cannot be used by the STAN device for ST waveform analysis. The lack of ST waveform detection by the STAN device is much less severe during the baseline period (14% ST waveform loss). The loss of ST waveform detection by the STAN device during UCO can also not be explained by a loss of fetal ECG loss or by fetal bradycardia (fetal HR <50 bpm). This suggests that the inability to detect ST waveform changes is within the STAN device. In a previous paper we calculated waveform-based ECG markers, during normoxemia and hypoxemia. Also, the absence of STAN-events due to poor signal quality in human fetuses associated with metabolic acidosis has been reported in literature [25, 26]. We conclude that improvement of the algorithm might result in more sensitive event warning.

Identifying causes for the high false event rate produced by STAN and the reduced ST waveform assessment during UCO is hampered since both the signal processing and the signal analysis of the STAN device are not available in public domain. One possible cause for poor performance of the STAN device lies in the hypoxia-induced changes in ST waveform. By cross-referencing input ECG, we found that in several subjects T waves initially became more negative during hypoxia, which was also observed by others [4, 14]. We speculate that the algorithm for STAN does not take the possibility of T wave decreases into account. If this were the case, it would negatively affect STAN performance because part of the ST waveforms would not be assessed correctly. A second possibility for a poor performance of the STAN device is related to the fetal electrical heart axis. The orientation of the fetal electrical heart axis differs greatly among fetuses [27]. The electrical heart axis affects the height of the T/QRS baseline and therefore the incidence of ST events. Since the height of the T/QRS baseline is irrespective of fetal condition [28] this should be taken into account in fetal monitoring with ST analysis. A recent study showed a significant increment of ST events with increasing height of the initial T/QRS baseline, which suggests that the orientation of the fetal electrical heart axis affects the height of the T/QRS baseline, and therefore the incidence of ST events [29].

The current study with its fetal sheep model of complete UCO has several limitations. First, though a complete UCO does result in a period of hypoxia followed by ischemia, it is not a reflection of the more common clinical scenario of intermittent or milder hypoxia-ischemia. However, the objective of our study was to first study accuracy of the STAN device under more discriminating circumstances of normoxemia and severe global hypoxia-ischemia. Second, UCO resulted immediately in a severe bradycardia. In clinical practice, in case of a prolonged bradycardia detected by CTG, it is clear that an intervention is indicated irrespective of the occurrence of ST events. Third, SDNN as a proxy for fetal wellbeing or compromise is only a crude estimate on the beat-to-beat time scale.

In conclusion, the performance of the STAN device in clinical practice is potentially limited by high false negative and high false positive STAN-event rates and loss of ST waveform assessment capacity during severe hypoxemia. Improving the STAN monitor to handle these issues may help the diagnostic value.
